# Plant Variety Selection Using Interaction Classes Derived From Factor Analytic Linear Mixed Models: Models With Independent Variety Effects

**DOI:** 10.3389/fpls.2021.737462

**Published:** 2021-09-09

**Authors:** Alison Smith, Adam Norman, Haydn Kuchel, Brian Cullis

**Affiliations:** ^1^Centre for Biometrics and Data Science for Sustainable Primary Industries, School of Mathematics and Applied Statistics, National Institute for Applied Statistics Research Australia, University of Wollongong, Wollongong, NSW, Australia; ^2^Australian Grain Technologies, Roseworthy, SA, Australia

**Keywords:** multi-environment trials, plant breeding, crop variety evaluation, linear mixed models, factor analytic linear mixed models, variety by environment interaction

## Abstract

A major challenge in the analysis of plant breeding multi-environment datasets is the provision of meaningful and concise information for variety selection in the presence of variety by environment interaction (VEI). This is addressed in the current paper by fitting a factor analytic linear mixed model (FALMM) then using the fundamental factor analytic parameters to define groups of environments in the dataset within which there is minimal crossover VEI, but between which there may be substantial crossover VEI. These groups are consequently called interaction classes (iClasses). Given that the environments within an iClass exhibit minimal crossover VEI, it is then valid to obtain predictions of overall variety performance (across environments) for each iClass. These predictions can then be used not only to select the best varieties within each iClass but also to match varieties in terms of their patterns of VEI across iClasses. The latter is aided with the use of a new graphical tool called an iClass Interaction Plot. The ideas are introduced in this paper within the framework of FALMMs in which the genetic effects for different varieties are assumed independent. The application to FALMMs which include information on genetic relatedness is the subject of a subsequent paper.

## 1. Introduction

Plant breeding multi-environment trials (METs) comprise series of variety trials conducted at a range of geographic locations and typically across several years (synonymous with seasons). They are an important component of identifying superior varieties as they allow an assessment of variety by environment interaction (VEI), that is, the differential performance of varieties in response to a change in environment. It is widely known that there are numerous advantages in analyzing MET datasets using a linear mixed model (LMM) approach in which a factor analytic (FA) variance structure is assumed for the variety effects in individual environments (see Smith et al., 2005, 2021; Gogel et al., 2018, for example). Key benefits are the ease with which incomplete data (not all varieties grown in all environments) can be handled, the ability to appropriately account for individual trial designs and the ability to include information on genetic relatedness, either through ancestral (pedigree) or genomic (marker) data. Furthermore, the FA component of the model consistently provides a good fit to the data and allows quantification and interpretation of VEI. All of this is achieved using a one-stage approach in which a single statistical analysis is conducted using the individual plot data combined across environments. This can be contrasted with two-stage approaches in which variety means from the separate analysis of each environment (stage 1) are used as “data” in a subsequent MET analysis (stage 2). There are inherent efficiency losses with two-stage approaches, even when adequate models are used in each stage (Gogel et al., 2018). Typically, further losses are incurred because two-stage approaches often employ simplistic models that rarely provide a good fit to the data. The clear benefits of the one-stage factor analytic linear mixed model (FALMM) approach has led to its use in the majority of Australian plant breeding programs and in the Australian National Variety Trials system (GRDC, 2021).

The fundamental information for variety selection from an FALMM are the predictions of the variety effects for individual environments (VE effects), where environments are defined to be the combinations of the geographic locations and years of the trials present in the dataset. For any given environment, the variety predictions are an accurate reflection of how the varieties performed in the environmental conditions that occurred at that particular location and in that particular year. Although this historical and specific perspective may be of interest, it is more likely that breeders and growers are concerned more generally with the performance of varieties across a range of environments that may be encountered in the future and reflect the target population of environments (see Cooper and Fox, 1996; Chenu et al., 2011, and references there-in). What is required, therefore, are meaningful summaries of the variety predictions across the environments in the dataset.

Smith and Cullis (2018) focussed on this problem, noting that the FALMM “out-performs others in terms of the model fitting component of a MET analysis but it has failed to deliver on the prediction component, in the sense of providing concise information to aid with variety selection.” They made a significant contribution toward the latter with their Factor Analytic Selection Tools (FAST) which included measures of variety performance and stability across all environments in the dataset. These measures were derived using the fact that a factor analytic model of order *k* (denoted FA*k*) for the VE effects, has a similar appearance to a multiple regression of the VE effects on *k* environment covariates (called loadings) and with separate slopes (called scores) for individual varieties. Unlike a multiple regression, however, both the covariates and the slopes are unknown. In the FALMM, the loadings are specified as variance parameters and the scores as random effects. Thus, the analysis provides residual maximum likelihood (REML) estimates of the loadings and empirical best linear unbiased predictions (EBLUPs) of the scores. As a post-processing step, the estimated loadings are rotated to a principal component solution, that is, such that the first rotated estimated loading accounts for the maximum amount of covariance in the VE effects, the second accounts for the next greatest amount and is orthogonal to the first, and so on (Smith et al., 2001; Smith and Cullis, 2018).

Given this framework, Smith and Cullis (2018) noted that the (rotated) estimated loadings for the first factor are often all positive in which case they represent a weighted average of all environments. Higher order factors are typically “bipolar” (Lawley and Maxwell, 1971), that is they have positive loadings for some environments and negative loadings for the remainder, so represent contrasts between environments. With this scenario, the first factor reflects overall variety performance combined with scale related (non-crossover) VEI and higher order factors reflect crossover VEI. Smith and Cullis (2018) therefore defined the overall performance (OP) for a variety using the EBLUP of the first factor score. Variety stability was defined as a function of the EBLUPs of the scores for all other (higher order) factors and this quantified the amount of crossover VEI exhibited by individual varieties.

The paradigm of the first factor representing a generalized variety main effect (defined as OP) and higher order factors representing crossover VEI is plausible and occurs often in practice. However, it raises the standard problem that exists in a factorial experiment, namely whether there is any sense or validity in examining main effects in the presence of interaction. The underlying statistical issues of marginality and “uninteresting hypotheses” have been discussed at length in Nelder (1977) and Nelder (1994). Venables (2000) points out that “If there is an interaction between factors A and B, it is difficult to see why the main effects for either factor can be of any interest, since to know what the effect of changing an A-level on the response will be depends on which B-level is in force.” Thus, in the FALMM context, variety OP may not be the most useful measure on which to base selection unless the first factor in an FA*k* model not only contains all positive estimated loadings, but also accounts for a large percentage of the total variance of the VE effects. If this is not the case, then crossover VEI in the dataset is non-ignorable and needs to be considered when making variety selection decisions.

In this paper we address the issue of summarizing variety performance in the presence of interaction by defining groups of environments within which crossover VEI is minimal. This is achieved using the fundamental parameters of the FA model and the characteristic that a bipolar factor represents a contrast between two sets of environments. Groups of environments formed on the basis of the signs of their estimated loadings in individual factors have the property that crossover VEI is minimized between environments in the same group but may be substantial between environments in different groups. The groups will therefore be called “interaction classes” (iClasses). It is then appropriate and meaningful to apply FAST separately to each iClass to aid with variety selection decisions.

In order to clearly elucidate the concepts of iClasses, the current paper will consider the simplest form of an FALMM in which the VE effects are assumed to be independent between varieties. Historically, this was the starting point for FALMMs (see Smith et al., 2001) and the model was applied to plant breeding MET datasets for all stages of selection and was also used for crop variety evaluation datasets. More recently, however, it has been shown there are substantial gains, particularly for early stage selection, in using an FALMM in which information on genetic relatedness (either ancestral or genomic) is included (Oakey et al., 2007; Beeck et al., 2010; Cullis et al., 2010; Smith et al., 2021). In terms of the iClass approach, FALMMs of this form have additional issues to consider and these will be discussed in a subsequent paper.

The paper is arranged as follows. In section 2 a motivating example comprising late stage wheat variety trials is described. Section 3 outlines the FALMM used in this paper which involves a modification to the variance assumptions previously used for the variety scores. Section 4 commences with a re-cap of the factor analytic selection tools proposed by Smith and Cullis (2018) then outlines the new approach of iClasses. The methods are applied to the motivating example in sections 5, 6 provides some concluding remarks.

## 2. Motivating Example

The MET dataset considered in this paper was constructed in order to demonstrate the iClass approach within the framework of an FALMM in which the VE effects are assumed independent between varieties. We stress that it is for illustrative purposes only.

The dataset comprised late-stage variety trials conducted by an Australian Grain Technologies (AGT) wheat breeding program over the period 2014–2017. The trials corresponded to the final two stages of testing in the program, namely stages 3 and 4 (S3 and S4) and were grown in Western Australia (WA), South Australia (SA), Victoria (Vic) and New South Wales (NSW). The aim of the analysis is to identify superior varieties amongst the 96 tested in S4 trials in 2017.

Across the full dataset there were 73 environments (trial location by year combinations, see Table 1) and 622 varieties. The 2017 data were balanced in the sense that all 96 varieties were grown in all 18 environments. The connectivity between 2017 trials and the earlier years is shown in Table 2. Of the 96 varieties tested in S4 in 2017, 27 were represented in all 4 years of this dataset; 12 were in the 3 years 2015, 2016, and 2017; one was in the 3 years 2014, 2015, and 2017; 26 were in the 2 years 2016 and 2017 and 30 appeared in 2017 alone.

**Table 1:** Environment summary information: numbers of (co-located) trials, plots, and varieties; mean yield (t/ha).

**Environment**	**Trials**	**Plots**	**Varieties**	**Mean yield**
14L01	3	624	320	3.58
14L03	2	384	236	3.24
14L04	3	624	320	3.27
14L05	2	408	191	1.52
14L06	3	576	320	1.73
14L07	3	576	320	2.38
14L08	2	408	191	1.41
14L09	3	576	320	2.19
14L10	2	408	191	1.07
14L12	3	576	320	3.15
14L13	2	504	236	2.67
14L14	2	384	236	3.46
14L15	3	576	320	1.69
14L16	2	384	236	1.86
14L17	3	624	320	4.14
14L18	2	408	191	1.59
14L19	2	384	236	2.84
14L20	2	504	236	2.42
14L21	2	504	236	5.61
14L23	2	504	236	5.05
15L01	2	468	214	3.07
15L02	2	372	214	1.51
15L03	2	372	214	4.33
15L04	2	468	214	2.89
15L05	1	228	114	3.38
15L06	2	372	214	2.55
15L07	2	372	214	1.95
15L08	1	228	114	3.43
15L10	1	228	114	3.66
15L11	2	372	214	4.46
15L12	2	372	214	3.06
15L13	2	468	214	1.84
15L14	2	372	214	4.23
15L17	2	468	214	3.50
15L18	1	228	114	1.45
15L19	2	372	214	2.83
15L20	2	468	214	1.64
15L21	2	468	214	4.14
15L22	2	468	214	3.82
15L23	2	468	214	3.46
16L01	2	528	216	2.05
16L02	2	456	216	4.72
16L03	2	456	216	6.42
16L04	2	528	216	4.40
16L05	1	288	112	3.73
16L08	1	288	112	3.82
16L10	1	288	112	2.03
16L11	1	288	112	2.50
16L14	1	288	112	6.16
16L17	2	528	216	2.46
16L18	1	288	112	1.97
16L19	2	456	216	5.37
16L20	2	528	216	3.69
16L21	2	528	216	6.95
16L23	2	528	216	7.42
17L01	1	288	96	3.46
17L02	1	288	96	3.76
17L04	1	288	96	2.17
17L05	1	288	96	1.98
17L06	1	288	96	1.19
17L07	1	288	96	3.29
17L08	1	288	96	1.33
17L10	1	288	96	1.48
17L11	1	288	96	4.41
17L12	1	288	96	3.25
17L13	1	288	96	5.28
17L15	1	288	96	1.93
17L18	1	288	96	1.07
17L19	1	288	96	3.35
17L20	1	288	96	3.50
17L21	1	288	96	5.70
17L22	1	288	96	2.47
17L23	1	288	96	5.14

*Environment name comprises the last two digits of the year of testing (2014–2017) and a code for the trial location (L01 - L23)*.

**Table 2:** Variety connectivity matrix across years: diagonal elements are numbers of varieties grown in individual years; off-diagonal elements are numbers in common between pairs of years.

	**2014**	**2015**	**2016**	**2017**
2014	320	82	45	28
2015	82	214	76	40
2016	45	76	216	65
2017	28	40	65	96

In 2017, each environment involved a single (S4) field trial whereas many of the environments in earlier years encompassed multiple trials, called co-located trials (Smith et al., 2021). These arose due to the conduct of both S3 and S4 trials at an environment (see Table 1). In our context a field trial is a physical block of plots onto which a valid experimental design (with replication and randomization) is imposed. The full dataset included 126 trials, each of which comprised a two-dimensional arrangement of plots indexed by rows and columns. Trials had either 12 or 24 columns and the number of rows ranged from 10 to 24 for trials with 12 columns and from 7 to 12 for trials with 24 columns. The number of varieties per trial ranged from 91 to 139 with a median of 110. In the majority of trials (103 of the 126), varieties were tested with replication (typically with two or three replicate plots). The remaining 23 trials used partially replicated designs (Cullis et al., 2006) in which some varieties were tested without replication (that is, a single plot for each) and others were tested using two replicate plots. On average, 25% of varieties within these trials had two replicate plots. Blocking was employed in the majority of trials with either two or three blocks. The blocks were aligned with columns or rows, or sometimes both columns and rows (corresponding to blocking in two directions).

## 3. Statistical Methods

It is assumed that the MET dataset comprises *p* environments, each of which may constitute a single trial or may encompass multiple (co-located) trials. Let ***y***_*j*_ denote the *n*_*j*_−vector of data for the *j*^*th*^ environment, *j* = 1…*p*. We then let ***y*** denote the *n*−vector of data combined across all environments in the MET, so write $y={\left(y_{1}^{⊤},y_{2}^{⊤},\ldots ,y_{p}^{⊤}\right)}^{⊤}$. Note that $n=\sum_{j=1}^{p}n_{j}$. The linear mixed model for ***y*** can be written as

(1) $$\begin{array}{r} y=X\tau +Z_{g}u_{g}+Z_{p}u_{p}+e \end{array}$$

where **τ** is a vector of fixed effects with associated design matrix ***X***; ***u***_***g***_ is the vector of random genetic effects with associated design matrix ***Z***_***g***_; ***u***_***p***_ is a vector of random non-genetic (or peripheral) effects with associated design matrix ***Z***_***p***_ and $e={\left(e_{1}^{⊤},e_{2}^{⊤},\ldots ,e_{p}^{⊤}\right)}^{⊤}$ is the combined vector of residuals from all environments. The vector of fixed effects includes mean parameters for individual environments. The vector of random peripheral effects includes effects associated with the designs of individual trials within environments. The variance matrix for ***u***_***p***_ is typically given by $G_{p}={⊕}_{i=1}^{b}\sigma_{p_{i}}^{2}I_{q_{i}}$ where *b* is the number of components in ***u***_***p***_ and *q*_*i*_ is the number of effects in (length of) ***u***_***p***_*i*__.

### 3.1. Variance Models for Genetic Effects

The random genetic effects comprise the variety effects nested within environments, and will be referred to as the VE effects. If we let *m* denote the total number of unique varieties across all environments, then the vector ***u***_***g***_ has length *mp*. We assume this is ordered as varieties within environments. In this paper, the nature of the MET dataset is such that no information on relationships between varieties is included in the analysis. This is the subject of a subsequent paper. Thus, it is assumed that

(2) $$\begin{array}{r} \text{var}\left(u_{g}\right)=G_{e}⊗I_{m} \end{array}$$

where ***G***_***e***_ is a *p* × *p* symmetric positive (semi)-definite matrix that will be referred to as the between environment genetic variance matrix. The matrix ***I***_*m*_ is an *m* × *m* identity matrix.

#### 3.1.1. Factor Analytic Model for VE Effects

A factor analytic model of order *k*, denoted FA*k*, is assumed for the VE effects and is written as

(3) $$\begin{array}{r} u_{g}=\left(\Lambda ⊗I_{m}\right)f+\delta \end{array}$$

where **Λ** is the *p* × *k* matrix of environment loadings for individual factors; ***f*** is the *mk*−vector of variety scores (ordered as varieties within factors) and **δ** is the *mp*−vector of VE lack of fit effects. It is assumed that ***f*** and **δ** are independent and distributed as multivariate Gaussian with zero means and variance matrices given by

(4) $$\begin{array}{r} \text{var}\left(f\right)=D⊗I_{m}\;\text{and var}\left(\delta \right)=\Psi ⊗I_{m} \end{array}$$

where ***D*** is a *k* × *k* symmetric positive (semi)-definite matrix that will be referred to as the factor score variance matrix and **Ψ** is a *p* × *p* diagonal matrix with elements referred to as specific variances. These assumptions lead to a variance matrix for the VE effects of the form

(5) $$\begin{array}{r} \text{var}\left(u_{g}\right)=\left(\Lambda D\Lambda^{⊤}+\Psi \right)⊗I_{m} \end{array}$$

so that the between environment genetic variance matrix is given by $G_{e}=\Lambda D\Lambda^{⊤}+\Psi$.

It is important to note that constraints must be imposed on **Λ** and ***D*** to ensure a unique solution. This is required for both estimation and interpretation but different constraints may be chosen for each purpose. In terms of interpretation, Smith and Cullis (2018) adopt the constraints that (a) the factor scores are independent with unit variance so that ***D*** is an identity matrix and (b) the loadings are such that **Λ**^⊤^**Λ** is a diagonal matrix with elements written in decreasing order. Although the constraints in (a) are commonly applied in the field of factor analysis, they lead to atypical properties when the FA model is embedded within a LMM. Typically, the random effects in a LMM have variances on a scale given by the square of the units of the trait under study. Their associated design matrices are free of this scale. The constraints of unit variance for the factor scores leads to the opposite scenario. Thus, to maintain consistency across all sets of random effects in the FALMM, we use a variation in which we assume that (a) the factor scores are independent so that ***D*** is a diagonal (non-identity) matrix with elements *d*_*r*_ (*r* = 1…*k*) and furthermore these are written in decreasing order and (b) the loadings are such that **Λ**^⊤^**Λ** is an identity matrix (that is, the columns of **Λ** are orthonormal vectors). The constraints required for estimation will be discussed in section 3.3.

It is instructive to write the FA*k* model for the VE effects in expanded form. We therefore write **Λ** = [**λ**_1_, …, **λ**_*k*_] where **λ**_*r*_ is the *p*−vector of environment loadings for factor *r* and write $f={\left(f_{1}^{⊤},\ldots ,f_{k}^{⊤}\right)}^{⊤}$ where ***f***_*r*_ is the *m*−vector of variety scores for factor *r*. The model in Equation (3) can then be written as

(6) $$\begin{array}{r} u_{g}=\left(\lambda_{1}⊗I_{m}\right)f_{1}+\left(\lambda_{2}⊗I_{m}\right)f_{2}+\ldots +\left(\lambda_{k}⊗I_{m}\right)f_{k}+\delta \end{array}$$

This has the appearance of a multiple regression with *k* terms in which the independent variables are the environment loadings (**λ**_*r*_), and there are separate slopes for individual varieties which are given by the variety scores (***f***_*r*_). The percentage of genetic variance accounted for by the *r*^*th*^ term (factor) is then given by

$$\begin{array}{l} v_{r}=100\times \text{tr}\left(\text{var}\left(\left(\lambda_{r}⊗I_{m}\right)f_{r}\right)\right)/\text{tr}\left(\text{var}\left(u_{g}\right)\right)\\ \;=100\times d_{r}/\text{tr}\left(G_{e}\right) \end{array}$$

and since the factor score variances, *d*_*r*_, are in decreasing order then so too are the variances accounted for, that is *v*_1_ > *v*_2_ > … > *v*_*k*_.

Following Smith and Cullis (2018) we define the common VE (CVE) effects as ***β*** = (**Λ** ⊗ ***I***_*m*_)***f*** so that ***u***_***g***_ = ***β*** + **δ**. The descriptor stems from the fact that these VE effects can be interpreted as fitted values in the FA model so represent sources of genetic covariance that are “common” to multiple (at least two) environments. In contrast, the lack of fit effects **δ** represent variation that is specific to individual environments so will hence-forth be called the specific VE (SVE) effects. In terms of the dataset under study, the CVE effects reflect repeatable sources of genetic variation where-as the genetic variation associated with the SVE effects is non-repeatable. As discussed in Smith and Cullis (2018), predictions of the CVE effects can be obtained for the complete two-way (variety by environment) table, irrespective of whether varieties were grown in an environment. This implies that it is reasonable to summarize CVE effects across any subset (or all) environments, a property that will be exploited in section 4.2. In contrast, in the absence of information on genetic relatedness, predictions of the SVE effects will be zero in cases where varieties were not grown in an environment. It is therefore not possible to summarize VE effects (which are the sum of the CVE and SVE effects) across environments without paying careful attention to the pattern of “missingness” in the variety by environment table. The VE effects may still be considered on an individual environment basis, but as discussed in section 1, we view these purely as a reflection of variety performance as it happened in the particular location and year of the environment concerned. Finally we note that if the percentage of variance accounted for by the FA model for an individual environment is high, then the predicted VE and CVE effects for the environment will be very similar (and equal in the case of 100% variance accounted for).

### 3.2. Variance Models for Residuals

The variance matrix for the residuals is given by var(***e***) = ***R*** and is assumed to be block diagonal, so that $R={⊕}_{i=1}^{p}R_{j}$ where ***R***_*j*_ = var(***e***_*j*_) is the variance matrix for the residuals for the *j*^*th*^ environment. In the LMM of Smith et al. (2001), spatial models are used for the residuals so that the matrices ***R***_*j*_ correspond to separable autoregressive processes (Cullis and Gleeson, 1991). Note that terms that reflect the experimental designs are also included in the model.

### 3.3. Model Fitting and Estimation

Every LMM in this paper was fitted using ASReml-R (Butler et al., 2017). The FA variance models were fitted as in Smith and Cullis (2018), that is, by splitting the VE effects into the CVE and SVE effects, each with their own variance structure. Thus, the two variance models were:

$$\begin{array}{l} \text{var}\left(\beta \right)=\left(\Lambda D\Lambda^{⊤}\right)⊗I_{m}\\ \text{var}\left(\delta \right)=\Psi ⊗I_{m} \end{array}$$

Implementation of the constraints discussed in section 3.1.1 is difficult and ASReml-R (Butler et al., 2017) uses simpler constraints, namely to set ***D*** = ***I***_*k*_ and, for *k* > 1, to set all the elements in the upper triangle of **Λ** to zero. We denote the loading matrix with these constraints as Λ^*^ and the associated vector of scores as f^*^. The original forms can be re-constructed using a rotation based on the singular value decomposition of Λ^*^, namely

$$\Lambda^{*}=UL^{1/2}V^{⊤}$$

where ***U*** and ***V*** are *p* × *k* and *k* × *k* orthonormal matrices such that the columns of ***U*** are the eigenvectors of Λ^*^Λ^*⊤^ and the columns of ***V*** are the eigenvectors of Λ^*⊤^Λ^*^. The matrix ***L*** is a *k* × *k* diagonal matrix with elements given by the eigenvalues of Λ^*^Λ^*⊤^, in decreasing order. We then form **Λ** as Λ^*^VL^−1/2^ (= ***U***) and ***D*** as ***L***. Finally the variety scores ***f*** are formed as $\left(L^{1/2}V^{⊤}⊗I_{m}\right)f^{*}$ so that var(***f***) = ***D*** ⊗ ***I***_*m*_ as required.

If the variance parameters are known, the random effects in the model may be predicted using best linear unbiased predictions (BLUPs) and the fixed effects may be estimated using best linear unbiased estimates (BLUEs). These are all obtained as solutions to the mixed model equations (MME) (Henderson, 1950). Of particular interest are the variety scores, ***f***, the BLUPs of which are given by

(7) $$\begin{array}{r} \tilde{f}=\left(D\Lambda^{⊤}⊗I_{m}\right){Z_{g}}^{⊤}Py \end{array}$$

where ***P*** = ***H***^−1^ − ***H***^−1^***X***(***X***^⊤^***H***^−1^***X***)^−^ ***X***^⊤^***H***^−1^ and $H=\text{var}\left(y\right)=Z_{g}G_{g}{Z_{g}}^{⊤}+Z_{p}G_{p}{Z_{p}}^{⊤}+R$. The matrix (***X***^⊤^***H***^−1^***X***)^−^ is any generalized inverse of (***X***^⊤^***H***^−1^***X***). Note that the BLUPs of ***f*** can also be expressed as

(8) $$\begin{array}{r} \tilde{f}=\left(L^{1/2}V^{⊤}⊗I_{m}\right){\tilde{f}}^{*} \end{array}$$

where ${\tilde{f}}^{*}=\left(\Lambda^{*⊤}⊗I_{m}\right){Z_{g}}^{⊤}Py$ are the BLUPs of f^*^.

The variance parameters are unknown, however, and are estimated using residual maximum likelihood (REML). These are then substituted into the MME which leads to empirical best linear unbiased estimates (EBLUEs) and empirical best linear unbiased predictions (EBLUPs) of the fixed and random effects, respectively. Note that if we denote the REML estimate of the loadings matrix from ASReml-R (Butler et al., 2017) as ${\hat{\Lambda }}^{*}$, we then use a singular value decomposition on this matrix to form $\hat{\Lambda }$ and $\hat{D}$. The EBLUPs of f^*^ are obtained directly from ASReml-R (Butler et al., 2017) and the EBLUPs of ***f*** are obtained using Equation (8) with ***L*** and ***V*** obtained from the singular value decomposition of ${\hat{\Lambda }}^{*}$. In the remainder of this paper BLUPs and EBLUPs will both be represented using the “tilde” notation and the distinction will be made in words. The code for fitting and summarizing the models is provided in the Supplementary Material.

## 4. Factor Analytic Selection Tools (FAST)

### 4.1. FAST of Smith and Cullis (2018)

In the case where all (or nearly all) the estimated loadings for the first factor are positive, Smith and Cullis (2018) defined the overall performance (OP) for variety *i* as

(9) $$\begin{array}{r} {\text{OP}}_{i}={\bar{\lambda }}_{1}{\tilde{f}}_{1i} \end{array}$$

where ${\bar{\lambda }}_{1}$ is the mean of the estimated loadings for the first factor. Smith and Cullis (2018) noted that OP could be viewed in terms of the first latent regression plot (LR1) for a variety, namely a scatter plot with the EBLUPs of the CVE effects as the *y*− axis and the estimates of the first factor loadings as the *x*− axis. The fitted regression line is also shown on this plot and has slope given by the EBLUP of the first factor score and an intercept of zero. OP is then the fitted value (point on the regression line) corresponding to an *x*−value of ${\bar{\lambda }}_{1}$. We note that it is also the mean of the fitted values for individual environments along this regression line. Smith and Cullis (2018) also used the first latent regression plot to define a measure of stability for each variety in terms of the “residual” sums of squares about the regression line. Thus, for variety *i* they defined stability as the root mean squared deviation (RMSD) which is given by

(10) $$\begin{array}{r} {\text{RMSD}}_{i}=\sqrt{\overset{p}{\underset{j=1}{\sum }}{\left({\tilde{\beta }}_{ij}-{\hat{\lambda }}_{1j}{\tilde{f}}_{1i}\right)}^{2}/p} \end{array}$$

Note that with our new constraints, this could also be written as $\sqrt{\sum_{r=2}^{k}{\tilde{f}}_{ri}^{2}/p}$.

### 4.2. Interaction Classes and Extensions of FAST

As discussed in section 1, the use of OP (a generalized variety main effect) in the presence of crossover VEI may be misleading. We propose identification of groups of environments, called interaction classes (iClasses), within which there is minimal crossover VEI. By definition, crossover VEI as modeled by the FALMM, is associated with bipolar factors. Hence a natural and simple approach for minimizing crossover VEI across all factors is to first map the estimated loadings for factor *r* (*r* = 1…*k*) to a categorical variable ***S***_*r*_ which has only two possible values for environment *j* (*j* = 1…*p*):

(11) $$\begin{array}{r} S_{rj}=\text{sign}\left({\hat{\lambda }}_{rj}\right)=\{\begin{array}{c} \text{“p” (positive) if }{\hat{\lambda }}_{rj}>0\\ \text{“n” (negative) if }{\hat{\lambda }}_{rj}<0 \end{array} \end{array}$$

iClasses are then formed from all possible combinations of the values (“p” or “n”) of the categorical variables ***S***_*r*_. Using the notation of Bailey (2008) this can be written as iClass = ***S***_1_ ∧ ***S***_2_ ∧ … ∧ ***S***_*k*_. Thus in an FA*k* model there are potentially 2^*k*^ iClasses and these can be labeled with a *k*-character code which is a “paste” of the possible values in ***S***_*r*_. For example, in an FA3 model there is a maximum of 8 iClasses and we denote the set of labels by Ω = (ppp, ppn, pnp, pnn, npp, npn, nnp, nnn). Note that not all 2^*k*^ iClasses may be represented in the dataset.

In this way, every environment is classified into one iClass. Within each iClass, the estimated loadings for any factor have the same sign for all environments so that none of the factors represent contrasts between environments. Thus, all factors, rather than just the first, can be used to obtain a measure of OP. This will be termed iClass overall performance (iClassOP). Given the regression interpretation of the FA model, a natural measure of iClassOP for each variety and iClass is the prediction at the mean values of the factor loadings for those environments in the iClass. Due to the manner in which the iClasses are formed, this is equivalent to the mean of the CVE effects for the variety across the environments in the iClass. We let ${\bar{\lambda }}_{r\omega }$ denote the mean of the loadings for factor *r* (*r* = 1…*k*) across the environments in iClass ω (ω ∈ Ω), that is

$$\begin{array}{l} {\bar{\lambda }}_{r\omega }=\underset{j\in \omega }{\sum }{\hat{\lambda }}_{rj}/n_{\omega } \end{array}$$

where *n*_ω_ is the number of environments in iClass ω and the sum is taken over those environments. We can then calculate the iClassOP for variety *i* in iClass ω as

(12) $$\begin{array}{r} \text{OP:}\omega_{i}=\overset{k}{\underset{r=1}{\sum }}{\bar{\lambda }}_{r\omega }{\tilde{f}}_{ri}\\ \;=\underset{j\in \omega }{\sum }{\tilde{\beta }}_{ij}/n_{\omega } \end{array}$$

In the same way that the LR1 plots provide a visual interpretation of OP and RMSD for the complete set of environments, an analogous set of plots can be drawn for individual iClasses. These will be termed iClass first latent regression (iClassLR1) plots. For a given variety, these show the EBLUPs of the CVE effects plotted against the estimates of the first factor loadings for those environments in the iClass. The fitted regression lines are also shown on these plots and have slopes given by the EBLUP of the first factor score and intercepts that depend on the predicted values for the higher order factors. Specifically the intercepts are the predictions at the mean of the loadings for factors 2…*k* for the iClass so are given by $\sum_{r=2}^{k}{\bar{\lambda }}_{r\omega }{\tilde{f}}_{ri}$. The key visual features of the full LR1 plots can be transferred to the iClassLR1 plots. Thus, iClassOP for a variety is the point on the regression line in the iClassLR1 plot that corresponds to the mean value of the first factor loadings for that iClass. iClassRMSD can be calculated in an obvious manner using the residual sums of squares about the iClassLR1 regression line.

## 5. Results

### 5.1. Non-genetic Effects and Spatial Variation

Although the genetic (VE) effects are of prime interest, we first consider other key components of the FALMM, namely the peripheral (non-genetic) effects for individual environments and the spatial models for the residuals for individual environments. The non-genetic effects fitted in these data included random effects for replicate blocks (as commensurate with the trial designs) and random row and column effects reflecting extraneous variation. In the case of environments with co-located trials, these peripheral random effects were nested within trials (so that random effects for trials within environments were also included in the model). The residuals for an environment were assumed to follow a two-dimensional (row by column) separable process (Cullis and Gleeson, 1991) in which the component correlation structures related either to an autoregressive process of order one or independence. In the case of environments with co-located trials, rows and columns were indexed within trials so that the spatial models were applied at the trial level but the parameters were constrained to be equal across the (co-located) trials. This is a pragmatic alternative to fitting a single spatial correlation structure that encompasses the entire environment (Cullis, pers comm). A summary of the non-genetic effects and spatial models fitted to the data is given in Table 3.

**Table 3:** Summary of non-genetic effects and spatial models for individual environments in the FALMM.

**Row**	**Column**			**Spatial**	**Number of**
**block**	**block**	**Row**	**Column**	**model**	**environments**
−	✓	✓	✓	ar1 × ar1	14
−	✓	−	✓	ar1 × ar1	13
−	−	✓	✓	ar1 × ar1	8
✓	✓	✓	✓	ar1 x ar1	7
−	✓	✓	−	ar1 × ar1	5
−	✓	✓	✓	id × ar1	5
−	✓	✓	✓	ar1 × id	3
−	−	−	✓	ar1 × ar1	2
✓	−	−	✓	ar1 × ar1	2
✓	✓	−	✓	ar1 × ar1	2
−	−	✓	✓	id × ar1	2
✓	✓	✓	✓	id × ar1	2
−	−	✓	−	ar1 × ar1	1
✓	✓	✓	−	ar1 × ar1	1
✓	−	✓	✓	ar1 × ar1	1
✓	✓	✓	✓	ar1 × id	1
−	✓	✓	−	id × ar1	1
−	✓	−	✓	id × ar1	1
−	✓	−	✓	id × id	1
−	−	✓	✓	id × id	1

*Non-genetic effects included random effects for blocks in the row and/or column dimension (Row and Column Block, respectively) and random Row and/or Column effects. Spatial models were all two-dimensional (column x row) with each dimension an autoregressive process of order one (ar1) or independence (id). Final column gives the number of environments for the given combination of non-genetic effects and spatial model and is listed in order of frequency of occurrence*.

### 5.2. Genetic Effects

In terms of the VE effects, an FA4 model was fitted and accounted for a total of 74.2% of the VE variance with the individual factors contributing 47.5, 16.1, 6.5, and 4.1% respectively. We comment that, in practice, we may aim for a larger total percentage which would require the fitting of higher order models. However, if we consider individual environments, the median percentage variance accounted for was 79.1% and only 6 environments had a percentage variance accounted for of less than 65% (see Table 4). Thus, the FA4 model has explained a high percentage of genetic variance for the majority of environments. For this reason and for ease of presentation we have used the FA4 model to demonstrate the iClass methodology.

**Table 4:** Summary of environment information from FA4 model fitted to VE effects: rotated REML estimates of loadings for each factor and percentage variance accounted for by all four factors.

	**Estimated loadings**					
**Environment**	**Factor 1**	**Factor 2**	**Factor 3**	**Factor 4**	**%vaf**	**iClass**
14L09	0.108	–0.008	–0.030	–0.014	75	pnnn
14L15	0.073	–0.013	–0.104	–0.022	73	pnnn
16L01	0.086	–0.059	–0.209	–0.174	70	pnnn
16L05	0.192	–0.223	–0.219	–0.248	85	pnnn
16L08	0.141	–0.102	–0.067	–0.008	82	pnnn
17L01	0.120	–0.040	–0.235	–0.109	90	pnnn
17L02	0.134	–0.046	–0.022	–0.012	72	pnnn
17L12	0.100	–0.046	–0.024	–0.064	95	pnnn
14L12	0.101	–0.014	–0.081	0.024	77	pnnp
15L08	0.087	–0.083	–0.129	0.120	70	pnnp
15L10	0.090	–0.210	–0.179	0.077	77	pnnp
16L03	0.111	–0.146	–0.031	0.291	71	pnnp
16L14	0.142	–0.057	–0.140	0.369	82	pnnp
16L21	0.157	–0.291	–0.062	0.133	70	pnnp
14L20	0.076	–0.019	0.153	–0.050	80	pnpn
15L02	0.054	–0.046	0.139	–0.001	83	pnpn
15L20	0.060	–0.061	0.095	–0.054	95	pnpn
16L02	0.206	–0.384	0.265	–0.255	86	pnpn
16L04	0.160	–0.219	0.022	–0.036	90	pnpn
16L10	0.066	–0.023	0.018	–0.060	74	pnpn
16L18	0.081	–0.009	0.112	–0.055	81	pnpn
17L20	0.121	–0.055	0.084	–0.173	78	pnpn
16L19	0.184	–0.099	0.061	0.059	65	pnpp
16L20	0.117	–0.144	0.097	0.001	79	pnpp
16L23	0.231	–0.234	0.186	0.277	62	pnpp
17L04	0.079	–0.005	0.137	0.001	85	pnpp
17L11	0.144	–0.113	0.141	0.181	77	pnpp
14L05	0.081	0.060	–0.082	-0.136	95	ppnn
14L07	0.143	0.149	-0.295	–0.074	90	ppnn
14L19	0.123	0.011	–0.108	–0.087	77	ppnn
15L06	0.077	0.011	–0.116	-0.082	52	ppnn
15L07	0.077	0.104	–0.031	–0.013	94	ppnn
15L11	0.121	0.107	–0.026	–0.107	31	ppnn
15L22	0.240	0.270	–0.095	–0.154	84	ppnn
16L11	0.183	0.026	–0.168	–0.034	29	ppnn
17L06	0.077	0.006	–0.120	–0.048	75	ppnn
17L07	0.182	0.030	–0.046	–0.134	68	ppnn
14L17	0.110	0.106	–0.041	0.128	75	ppnp
15L05	0.127	0.003	–0.153	0.051	74	ppnp
15L12	0.133	0.187	–0.079	0.022	78	ppnp
15L19	0.127	0.055	–0.169	0.035	92	ppnp
15L23	0.109	0.048	–0.072	0.001	85	ppnp
17L10	0.037	0.003	–0.131	0.000	98	ppnp
17L21	0.096	0.080	–0.101	0.175	69	ppnp
17L23	0.109	0.191	–0.067	0.213	97	ppnp
14L01	0.124	0.009	0.159	–0.000	80	pppn
14L03	0.090	0.028	0.087	–0.040	96	pppn
14L04	0.123	0.118	0.060	–0.046	84	pppn
14L06	0.052	0.019	0.035	–0.037	72	pppn
14L08	0.047	0.072	0.076	–0.121	73	pppn
14L10	0.056	0.057	0.002	–0.059	78	pppn
14L13	0.113	0.089	0.222	–0.151	88	pppn
14L14	0.111	0.018	0.140	–0.074	72	pppn
14L16	0.031	0.009	0.050	–0.002	33	pppn
14L18	0.053	0.000	0.050	–0.020	71	pppn
15L13	0.112	0.146	0.180	–0.100	74	pppn
16L17	0.085	0.043	0.128	–0.049	92	pppn
17L05	0.064	0.045	0.020	–0.013	80	pppn
17L15	0.074	0.036	0.016	–0.071	88	pppn
17L18	0.033	0.021	0.057	–0.044	91	pppn
17L19	0.101	0.066	0.077	–0.016	77	pppn
17L22	0.108	0.104	0.017	–0.147	78	pppn
14L21	0.134	0.236	0.148	0.091	84	pppp
14L23	0.171	0.146	0.116	0.085	84	pppp
15L01	0.119	0.094	0.042	0.007	85	pppp
15L03	0.076	0.157	0.079	0.100	72	pppp
15L04	0.097	0.117	0.122	0.077	86	pppp
15L14	0.076	0.120	0.033	0.254	79	pppp
15L17	0.088	0.076	0.001	0.034	80	pppp
15L18	0.045	0.009	0.067	0.053	69	pppp
15L21	0.094	0.170	0.120	0.021	63	pppp
17L08	0.034	0.038	0.021	0.017	81	pppp
17L13	0.185	0.025	0.029	0.158	88	pppp

*Final column shows iClass membership. Environments have been ordered alphabetically within iClasses*.

#### 5.2.1. Environment Loadings and Formation of iClasses

The (rotated) REML estimates of the loadings for individual environments are presented in Table 4. First note that all of the estimated loadings for the first factor are positive, indicating that this factor represents a weighted average of environments. The remaining factors are all bipolar so represent contrasts between environments. For example, the second factor represents contrasts between the first 27 listed environments in Table 4 and the remaining 46 environments. The signs of the individual estimated loadings were used to create the categorical variables ***S***_*r*_ (*r* = 1…4) and thence iClasses were formed as ***S***_1_ ∧ ***S***_2_ ∧ ***S***_3_ ∧ ***S***_4_ as described in section 4.2. Only 8 of the possible 2^4^ = 16 iClasses were present in the data and these corresponded to Ω = (pnnn, pnnp, pnpn, pnpp, ppnn, ppnp, pppn, pppp). The classification of environments into iClasses is shown in Table 4. For clarity, this table has been ordered on iClasses which highlights the groupings of environments whose estimated loadings have the same sign and thence the elimination of contrasts between environments within the same iClass.

**Figure 1:**
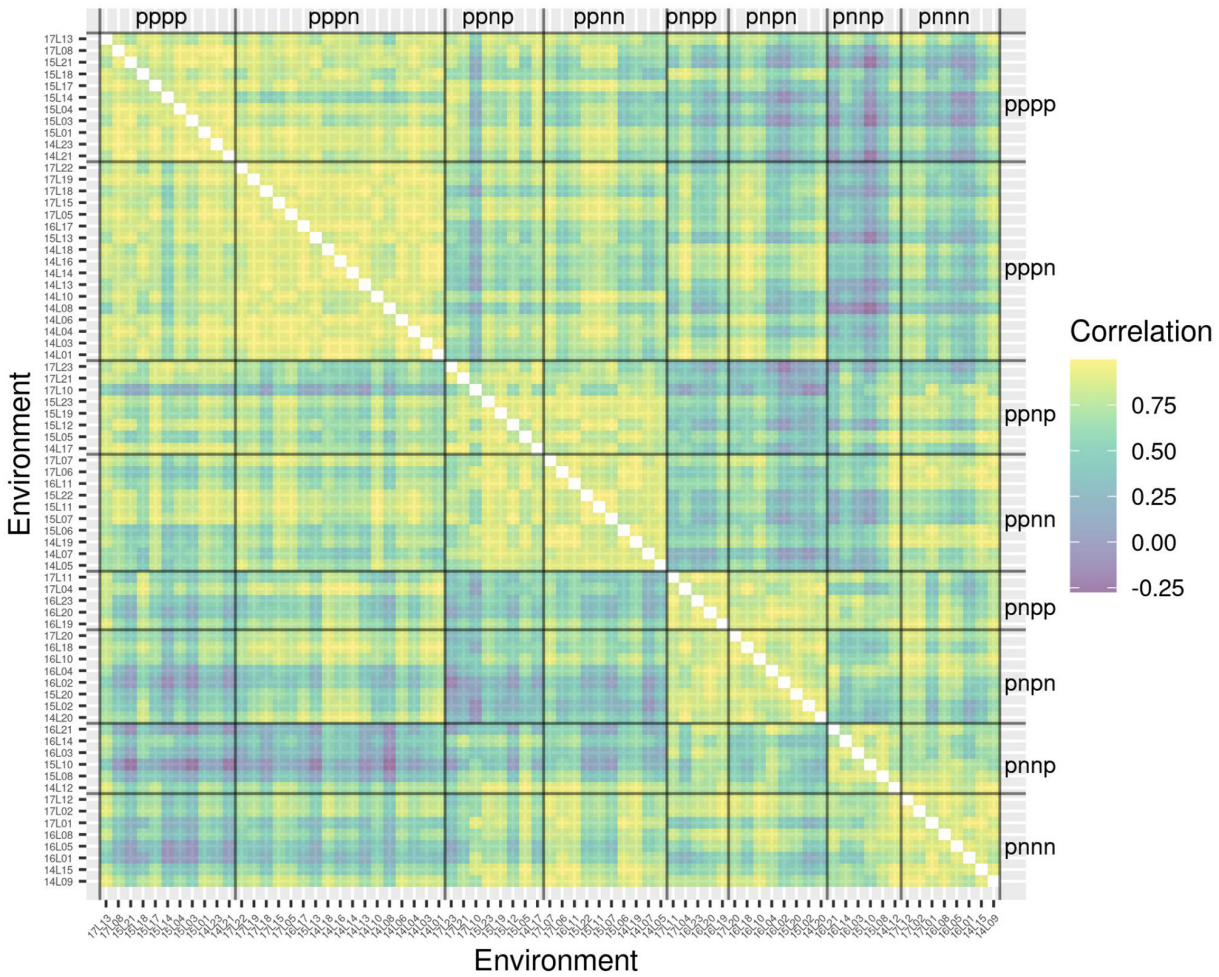
Estimated genetic correlations for CVE effects for all pairs of environments. Environments are ordered according to iClasses.

**Figure 2:**
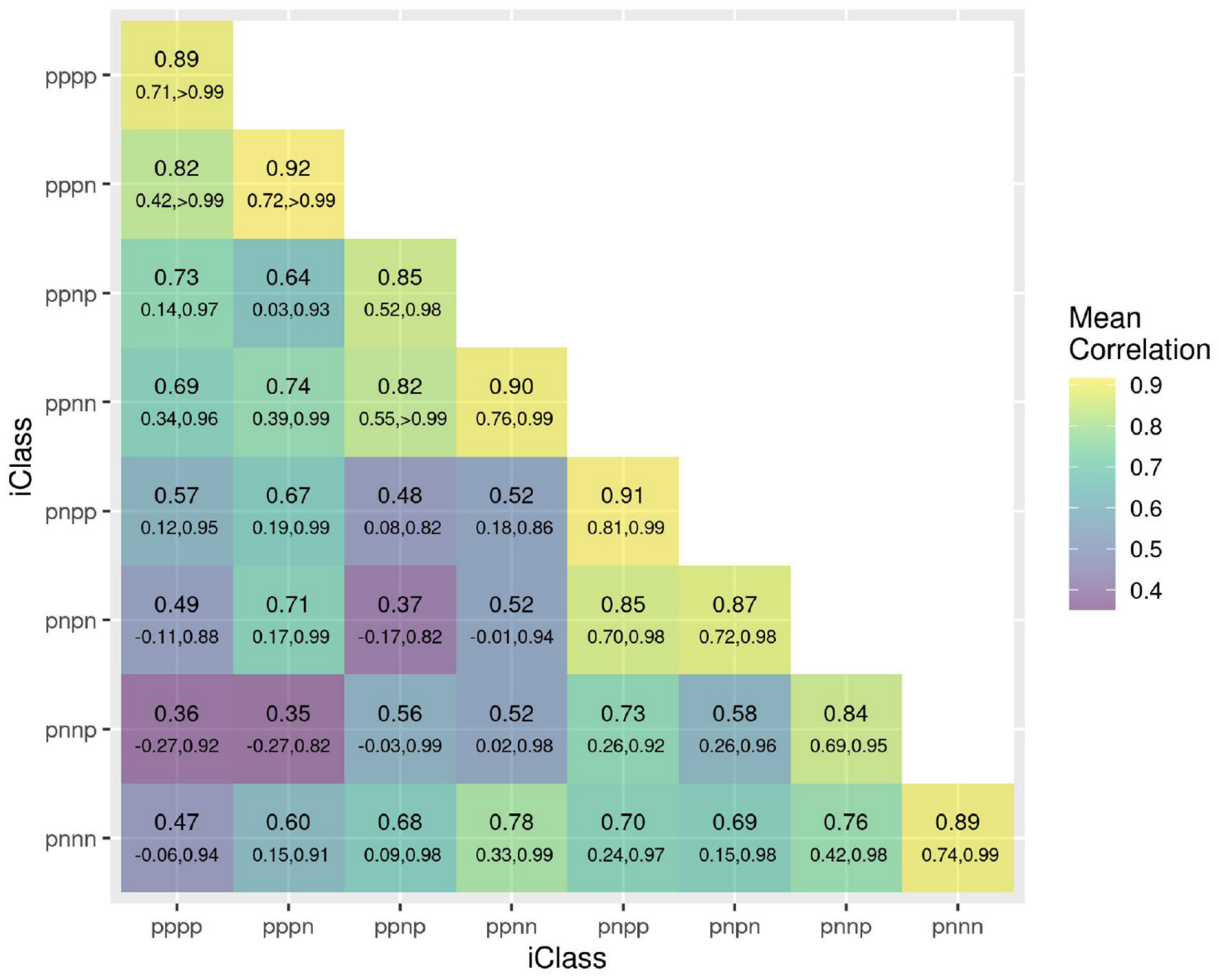
Estimated genetic correlations for CVE effects for all pairs of environments summarized on an iClass basis. The value listed uppermost in each cell is the mean of all pairwise estimated correlations between environments in the iClass; the values underneath are the minimum and maximum correlations between environments in the iClass. The color scale corresponds to the mean values.

The success of the iClasses in terms of minimizing crossover VEI can be assessed using the between environment genetic variance matrix for the CVE effects, that is, $\Lambda D\Lambda^{⊤}=G_{e}-\Psi$. Given that crossover VEI is synonymous with changes in variety rankings between environments, it is beneficial to first convert this to a correlation matrix. Figure 1 shows the full estimated correlation matrix as a heatmap, with the environments ordered according to iClasses. These estimated correlations have then been summarized on an iClass basis (see Figure 2). Both figures illustrate that there are strong correlations between all pairs of environments within each iClass. The mean pairwise correlations within iClasses range from 0.84 (iClass “pnnp”) up to 0.92 (iClass “pppn”). Thus, there is very little crossover VEI within iClasses. In terms of between iClass comparisons, there is substantial crossover VEI for some pairs of iClasses, as evidenced by low correlations. For example, the mean pairwise correlation between environments in iClass “pppn” and those in “pnnp” is only 0.35. Note that these iClasses differ in terms of every constituent bipolar factor (that is, factors 2–4). In general, the crossover VEI is least between those iClasses that differ only in the fourth factor and greatest for those that differ in the second factor. This is clearly seen in Figures 1, 2 since the iClasses are ordered such that the fourth factor changes the fastest and the second factor the slowest.

#### 5.2.2. Variety Scores and Performance Within iClasses

**Figure 3:**
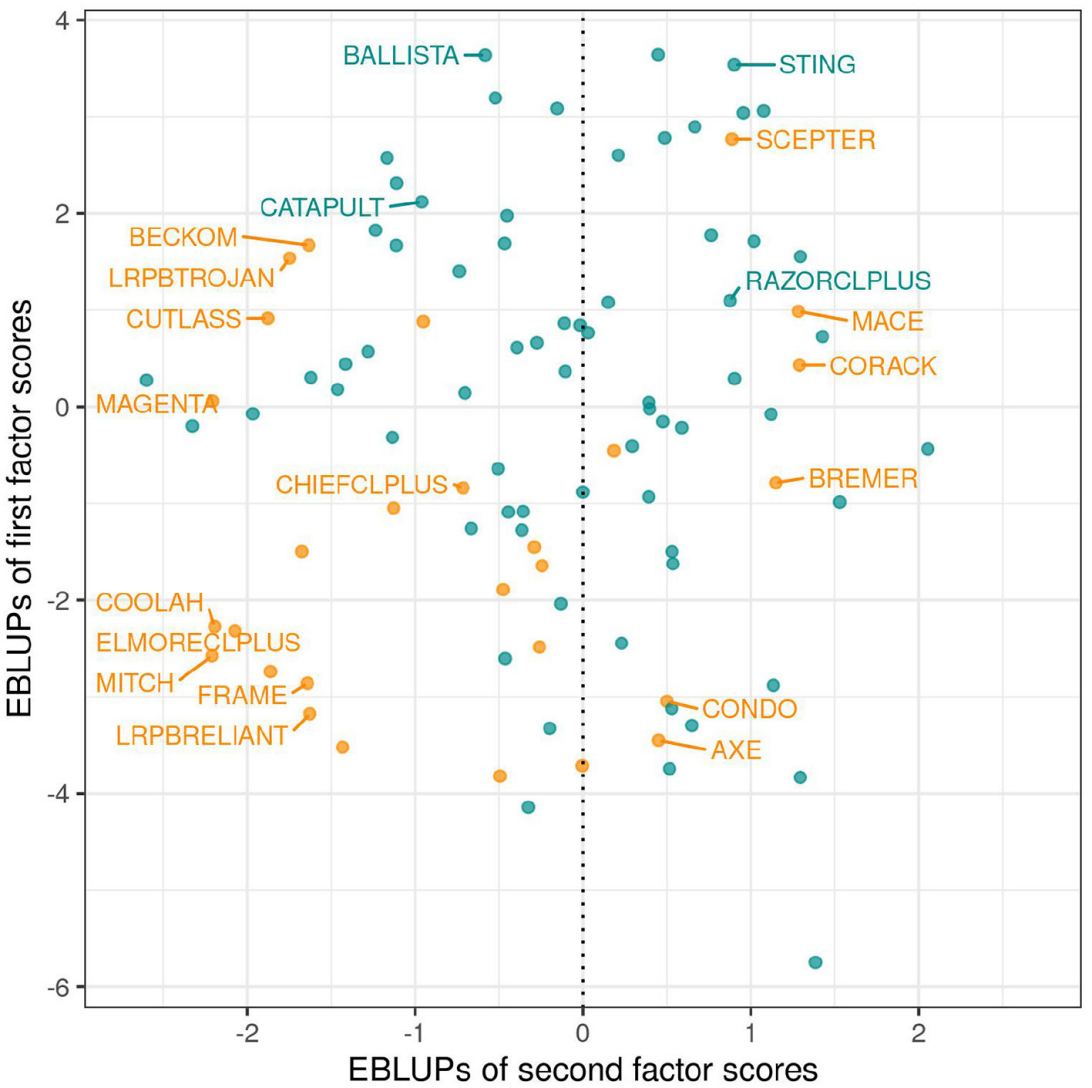
EBLUPs of first and second factor scores for the 96 varieties of interest. Points and labels are colored orange (check varieties) or blue (test varieties). Selected check varieties have been labeled as have four test varieties that have since been released commercially.

**Figure 4:**
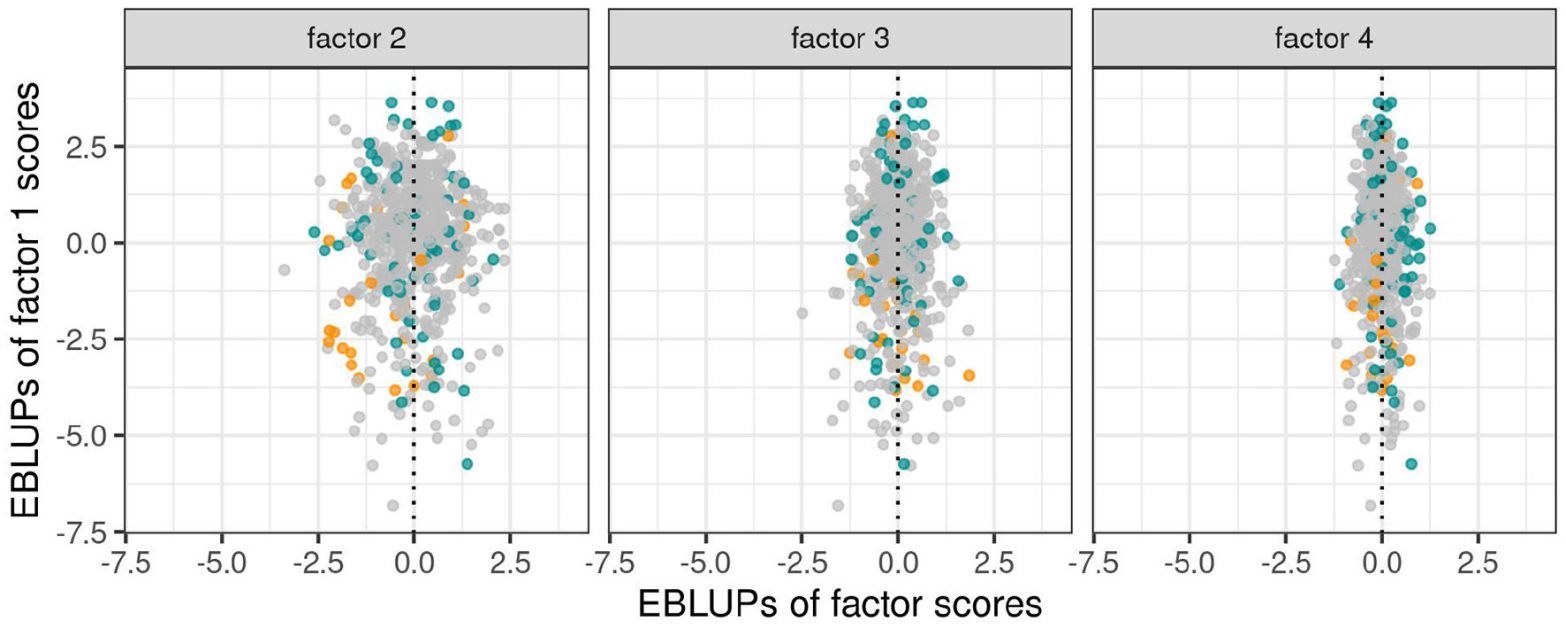
EBLUPs of first factor scores plotted against second, third and fourth factor scores for all varieties. Points are colored orange (check varieties), blue (test varieties of interest) or gray (remaining test varieties).

As discussed in section 3.1.1 the variety scores for a factor reflect the responses of the varieties to the environmental covariate implicit in the loadings for that factor. Varieties that have large and opposite signs for the scores associated with bipolar factors may exhibit substantial crossover VEI. It is therefore instructive to consider the EBLUPs of the variety scores and plots of the form given in Figure 3 are particularly helpful. This figure plots the EBLUPs of the first factor scores against the second for the 96 varieties of interest. On these plots the distinction has been made between check varieties (established commercial varieties) and test varieties (AGT breeding lines). Note that seven of these test lines have since been released commercially. Variety names have been included on these plots for selected varieties of interest. The new rotation in which the variance of the factor scores decreases from the first to the last factor is apparent in Figure 3 with the spread of the EBLUPs of first factor scores for these 96 varieties being far greater than for the second factor. This is more clearly seen in Figure 4 which contains the full series of score plots for all varieties in the dataset and in which all the axes have been drawn with the same limits.

In terms of interpretation of Figure 3 we first note that all the estimated loadings for the first factor were positive, so that the *y*−axis is a scaled version of the OP measure of Smith and Cullis (2018) as given in Equation (9). Thus, for the motivating example, the EBLUPs of the first factor scores are synonymous with OP. Of the named varieties in this dataset, BALLISTA, STING, and SCEPTER have the largest EBLUPs of the first factor scores and therefore the highest OP. Examination of the EBLUPs of the second factor scores in Figure 3 reveals that many of the named varieties with positive scores are early to mid maturing varieties, whereas those with negative scores are mid to late maturing varieties. The second factor therefore incorporates a maturity response but this does not provide a conclusive explanation.

**Figure 5:**
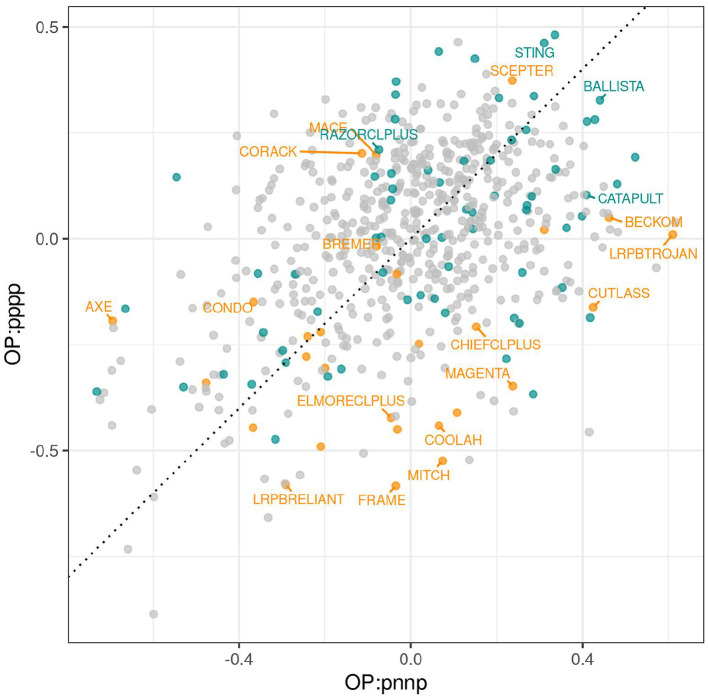
iClassOP for iClasses “pppp” and “pnnp” for all varieties. Points are colored orange (check varieties), blue (test varieties of interest) or gray (remaining test varieties). Selected check varieties have been labeled as have four test varieties that have since been released commercially.

Individual factors represent separate sources of VEI, and although interpretation of these sources can be illuminating, it is not essential for variety selection. A key point is that varieties with near zero scores for all bipolar factors are stable varieties whose relative yield performance will be similar across all environments in the dataset. In contrast, varieties with large positive or negative scores for some bipolar factors may exhibit large fluctuations in their relative yield performance. The crucial information, therefore, is the collective response of varieties to all factors and this is captured in iClassOP. The iClassOP for all varieties and each of the 8 iClasses was calculated using the regression prediction approach as in Equation (12). The values at which the predictions were made, that is, the mean of the estimated loadings for each factor for the iClass concerned are given in Table 5. To illustrate the existence of crossover VEI between iClasses, we have plotted iClassOP for “pppp” against “pnnp” in Figure 5. This pair of iClasses was chosen because the mean estimated genetic correlation between environments in the two iClasses was only 0.36 (see Figure 2). Figure 5 reveals some large changes in variety ranks between the two iClasses. For example, LRPBTROJAN is the highest ranking variety for iClass “pnnp” but is only mid ranking in “pppp”. The varieties STING and SCEPTER rank well for iClass “pppp” but less well for “pnnp”. The variety BALLISTA ranks near the top in both iClasses.

**Table 5:** Values of estimated loadings used in the predictions of OP for individual iClasses (means of loadings for each factor across the environments in the iClass).

					**Number of**
**iClass**	**Factor 1**	**Factor 2**	**Factor 3**	**Factor 4**	**environments**
pnnn	0.119	–0.067	–0.114	–0.082	8
pnnp	0.115	–0.133	–0.104	0.169	6
pnpn	0.103	–0.102	0.111	–0.085	8
pnpp	0.151	–0.119	0.125	0.104	5
ppnn	0.130	0.077	–0.109	–0.087	10
ppnp	0.106	0.084	–0.102	0.078	8
pppn	0.081	0.052	0.081	–0.058	17
pppp	0.102	0.108	0.071	0.081	11

*Final column shows the number of environments used to form the means*.

**Figure 6:**
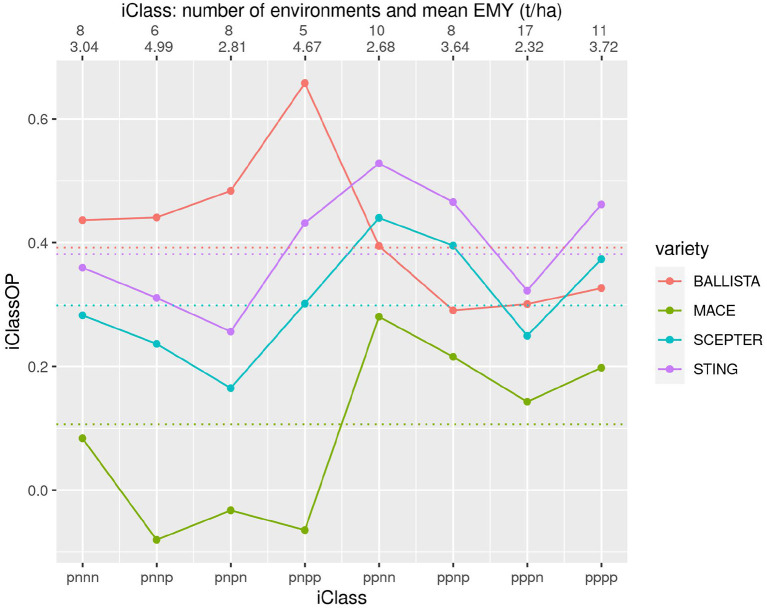
iClass interaction plot for the four varieties BALLISTA, MACE, SCEPTER and STING. The points and solid lines that join them correspond to iClassOP and the dotted horizontal lines correspond to the OP measure of Smith and Cullis (2018). The axis at the top shows the number of environments in each iClass and the mean of the associated environment mean yields.

**Figure 7:**
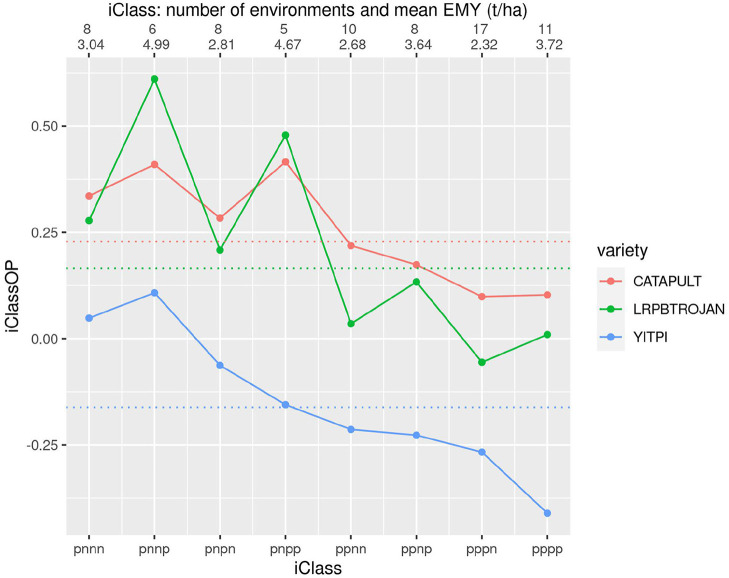
iClass interaction plot for the three varieties CATAPULT, LRPBTROJAN and YITPI. The points and solid lines that join them correspond to iClassOP and the dotted horizontal lines correspond to the OP measure of Smith and Cullis (2018). The axis at the top shows the number of environments in each iClass and the mean of the associated environment mean yields.

**Figure 8:**
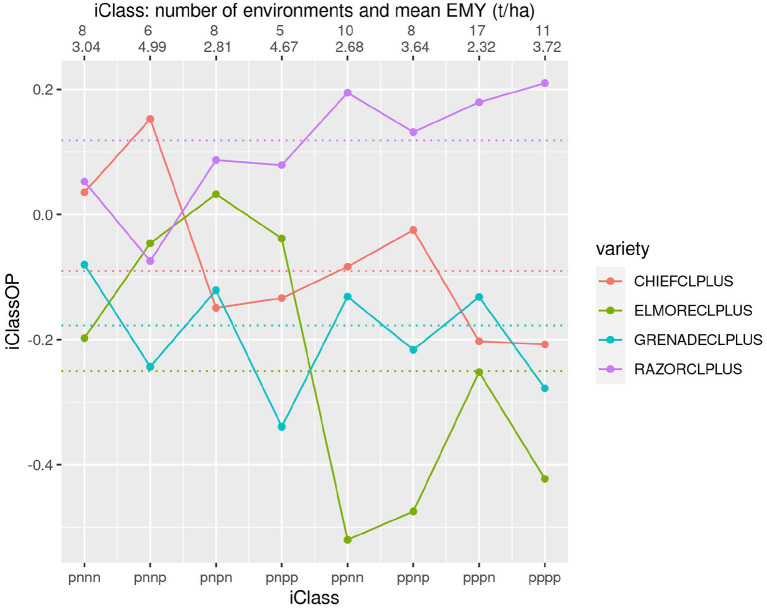
iClass interaction plot for the four varieties CHIEFCLPLUS, ELMORECLPLUS, GRENADECLPLUS and RAZORCLPLUS. The points and solid lines that join them correspond to iClassOP and the dotted horizontal lines correspond to the OP measure of Smith and Cullis (2018). The axis at the top shows the number of environments in each iClass and the mean of the associated environment mean yields.

To obtain a more detailed picture of crossover VEI we introduce a new graphical tool called an iClass Interaction Plot. This plots the iClassOP values for a selected set of varieties, with the points ordered on iClasses. Importantly this provides a meaningful ordering based on crossover VEI. This is because pairs of iClasses that differ only in the highest order factor (so reflect the least amount of between iClass crossover VEI) are located next to each other whereas pairs that differ in the lowest order factor (so reflect the greatest amount of crossover VEI) are located on opposite sides (left/right). Figures 6–8 are iClass Interaction Plots for groups of varieties chosen specifically as being of interest for grower comparisons. Note that these plots have been enhanced with information on the number of environments in each iClass and also the mean of the environment mean yields for the environments in each iClass.

Figure 6 contains the two new varieties BALLISTA and STING, that were released as alternatives to the two market leading varieties, MACE and SCEPTER. In terms of the newer varieties, Figure 6 shows that STING has an almost identical pattern of VEI to SCEPTER, and very similar to MACE, but with substantially improved yield performance. BALLISTA is high yielding across most environments but has a different pattern of VEI to the other three varieties, with a particular advantage in iClasses with an “n” in the second factor. The OP values based on Smith and Cullis (2018) are also shown on this figure as the dotted horizontal lines. Importantly this shows that the OP of the two new varieties is very similar. However, the use of OP alone would miss the key fact that these two varieties have a distinctly different pattern of VEI.

Figure 7 allows comparison of three longer season varieties including YITPI which is an older variety typically used as a benchmark. The plot shows the clear advantage of LRPBTROJAN and CATAPULT over YITPI. It also demonstrates that LRPBTROJAN and CATAPULT have similar patterns of VEI and that all varieties exhibit substantial VEI. The VEI for LRPBTROJAN, and to a lesser extent, CATAPULT, is linked to environment mean yield, with these varieties performing best in the iClasses with high environment mean yields (iClasses “pnnp” and “pnpp').

Figure 8 shows four varieties that incorporate Clearfield® technology. This figure shows some large differences in both yield performance and patterns of VEI, particularly for the comparison of RAZORCLPLUS with the rest.

## 6. Concluding Remarks

In this paper we have addressed the key issue of variety selection in the presence of VEI. This has been achieved within the framework of a (single-stage) FALMM which is widely regarded as the “gold standard” method of analysis for multi-environment trial data. The approach involves the formation of groups of environments, called iClasses, within which there is minimal crossover VEI. It is then statistically and biologically valid to identify the best varieties within each iClass. The idea of forming groups of environments with minimal VEI is not new. However, most previous attempts have been based on two-stage approaches, the disadvantages of which were discussed in section 1. Many methods are based on applying a singular value decomposition (SVD), typically to a matrix indexed by varieties and environments. This approach was first suggested by Kempton (1984) and numerous variations have since been proposed. Interpretation of VEI and groupings of environments are often based on a biplot (Kempton, 1984) which is a graphical display of the first two principal components from the SVD. We note there are similarities between the SVD method and the FA model (with the constraints described in section 3.1.1) in terms of the decomposition into components/factors of decreasing importance. A key difference with our approach is that this is achieved by embedding the FA model within the one-stage LMM. Additionally we use all factors to interpret VEI and form iClasses, rather than just the first two. In our experience, it is rare for the first two factors to account for a large percentage of VE variance so that interpretations based solely on two factors may be misleading. Finally, our method of forming groups does not involve the application of generic clustering methods to results extracted from the FALMM (such as the estimated genetic correlation matrix). Instead, iClasses are formed on the basis of the fundamental parameters of the FA model, namely the environment loadings, so are a direct consequence of the model used for analysis.

Comparisons of varieties within iClasses is achieved using an extension of the FA selection tools of Smith and Cullis (2018). In particular we have defined a measure of overall performance for varieties across the environments in each iClass (iClassOP) rather than the overall performance (OP) measure of Smith and Cullis (2018) which is taken across all environments in the dataset. We note also that the OP of Smith and Cullis (2018) requires all (or most) of the estimated loadings for the first factor to be positive whereas iClassOP has no such limitation. The existence of a mixture of positive and negative estimated loadings in the first factor merely creates additional iClasses.

The utility of the approach was clearly demonstrated in the application to the motivating example in which some large and important changes in the ranks of varieties between iClasses were revealed. This crossover VEI would not have been identified with the use of a single measure of overall performance, whether this be a simple average across environments or the more sophisticated generalized main effect embodied in the OP measure of Smith and Cullis (2018). The variety LRPBTROJAN provided a classic case of how meaningless a main effect can be in the presence of interaction. The iClass approach revealed that the performance of this variety differed substantially between environments. It was a top and near-top ranking variety in two iClasses but a mid-ranking variety in others. Another noteworthy, but more subtle example was the case of the two new varieties BALLISTA and STING. At the time of the commercial release of these varieties the iClass technology was not available and it was clear that the varieties had high and similar OP, suggesting that only one should be released. However, anecdotal evidence led the breeding company to believe that these varieties may have been differentially adapted to certain growing environments so the potentially risky decision was made to release both. Our analysis is in a sense a retrospective case study since these two varieties appeared as test varieties in the dataset. The iClass approach revealed that these two varieties were indeed generally high yielding but that they exhibited different patterns of VEI, thereby validating their joint release. Clearly the availablility of this type of information for release decisions would be invaluable as it aids in minimizing commercial risk. It would also be extremely useful for growers since their focus is typically the selection of new varieties that have improved yields but exhibit similar patterns of VEI to the varieties with which they are familiar.

To aid both breeders and growers with variety comparisons we have introduced iClass Interaction Plots. These plots display the iClassOP for individual iClasses for sets of varieties of interest. The plots have a similar appearance to those introduced in Smith et al. (2015) but the key difference is that the iClass Interaction Plots not only capture the major sources of crossover VEI in the data but also order this information in an enlightening manner.

It has been argued there are deficiencies in the use of an FALMM since it is only possible to obtain predictions of variety performance for the environments present in the MET dataset. What is required is knowledge of variety performance in the target population of environments (TPE). Many authors have therefore proposed the use of external environment variables as a means to extrapolate from a MET to the TPE. However, as Cooper and Fox (1996) comment, “there is generally limited knowledge of (i) important environmental variables and (ii) how these variables contribute to GxE interaction”. We believe that the use of an FALMM followed by the application of the iClass technology provides an alternative that helps bridge the gap between variety predictions for the environments in the MET and the TPE. This is because it enables identification of repeatable sources of VEI, together with their frequency of occurrence, within the MET. If the environments within the MET are a representative sample of the TPE then the iClass approach provides a platform for understanding and exploiting VEI in the TPE.

Finally we note that the MET dataset used in this paper corresponded to late-stage trials from a wheat breeding program. It is similar in structure to MET datasets from crop variety evaluation programs, in the sense that these typically include a wide range of environments so that crossover VEI may be expected. Additionally the varieties under investigation are either already commercially available or they are “elite” test lines. Within this framework we have demonstrated the success of the iClass approach in providing key information for commercial decisions by breeding companies and for grower selection decisions. The iClass approach will also be extremely beneficial for MET datasets that include early-stage plant breeding trials where the selection decisions relate to the promotion of lines for further testing. The optimum analysis of such data requires the use of information on genetic relatedness, either via ancestral or genomic data. The application of iClasses within this paradigm is the focus of a subsequent paper.

## Data Availability Statement

The data analyzed in this study is subject to the following licenses/restrictions: The dataset presented in this article is owned by Australian Grain Technologies. Requests to access these datasets should be directed to Adam.Norman@agtbreeding.com.au.

## Author Contributions

BC and AS conceived the ideas and developed the methodology. AN and HK provided the dataset, the motivation to develop the methodology and fruitful discussions about the results and presentation there-of. AS prepared the first draft of the manuscript. All authors contributed to manuscript revision, read, and approved the submitted version.

## Funding

AS was supported by the Grains Research and Development Corporation (GRDC) through the EssCargoT project (UW00010).

## Conflict of Interest

AN and HK are employed by Australian Grain Technologies. The remaining authors declare that the research was conducted in the absence of any commercial or financial relationships that could be construed as a potential conflict of interest.

## Publisher's Note

All claims expressed in this article are solely those of the authors and do not necessarily represent those of their affiliated organizations, or those of the publisher, the editors and the reviewers. Any product that may be evaluated in this article, or claim that may be made by its manufacturer, is not guaranteed or endorsed by the publisher.
