# Supplementary material for: Plant Variety Selection Using Interaction Classes Derived From Factor Analytic Linear Mixed Models: Models With Independent Variety Effects
Source: Front Plant Sci. 2021 Sep 9;12:737462. doi: 10.3389/fpls.2021.737462 (PMC8460066; doi:10.3389/fpls.2021.737462)
Supplement: Supplementary file 1 [file Data_Sheet_1.PDF]

1 In-house functions

First we include the functions we have written (in-house) that will be required to process the factor analytic linear mixed model output from ASReml-R:

```
vccmake.mfxlm <- function(obj=asr.sv,data=set1.df,Rownam='Row!cor',
                          Colnam='Col!cor', Resnam= "!R$",Envnam='Env',Trialnam='Trial') {
  temp <- obj$vparameters.table
  sigmas <- temp[grep(Resnam,temp$Component),]
  colphis <- temp[grep(Colnam,temp$Component),]
  rowphis <- temp[grep(Rownam,temp$Component),]
  ns <- dim(sigmas)[1]
  ncp <- dim(colphis)[1]
  nrp <- dim(rowphis)[1]
  temp.ss <- rbind(sigmas,colphis,rowphis)
  order <- temp$Component[temp$Component %in% temp.ss$Component]
  temp.ss$Trial <- sapply(strsplit(as.character(temp.ss$Component),split=c('_')), function(x) x[2])
  temp.ss$Trial <- sapply(strsplit(temp.ss$Trial,split=c('!')), function(x) x[1])
  temp.ss$Env <- factor(tapply(as.character(data[[Envnam]]),data[[Trialnam]],unique)[temp.ss$Trial])
  temp.ss$Partype <- factor(rep(c('sig','colphi','rowphi'),times=c(ns,ncp,nrp)))
  names(temp.ss)[1] <- 'Vparameter'
  if((ncp + nrp) ==0) {
    if(length(levels(temp.ss$Env))==1) {
      M <- vcm.lm(~1,data=temp.ss) # note: vcm.lm is an asreml function
    } else {
      M <- vcm.lm(~Env,data=temp.ss)
    }
  } else {
    if(length(levels(temp.ss$Env))==1) {
      M <- vcm.lm(~Partype,data=temp.ss)
    } else {
      M <- vcm.lm(~Env:Partype,data=temp.ss)
    }
  }
  attr(M,'assign') <- NULL; attr(M,'contrasts') <- NULL
  if(ns>0) {
    envmeans <- with(subset(temp.ss,Partype=='sig'),tapply(Value,Env,mean))
    temp.ss$Value[temp.ss$Partype=='sig'] <- envmeans[as.character(temp.ss$Env[temp.ss$Partype=='sig'])]
  }
  if(ncp>0) {
    envmeans <- with(subset(temp.ss,Partype=='colphi'),tapply(Value,Env,mean))
    temp.ss$Value[temp.ss$Partype=='colphi'] <- envmeans[as.character(temp.ss$Env[temp.ss$Partype=='colphi'])]
  }
  if(nrp>0) {
    envmeans <- with(subset(temp.ss,Partype=='rowphi'),tapply(Value,Env,mean))
    temp.ss$Value[temp.ss$Partype=='rowphi'] <- envmeans[as.character(temp.ss$Env[temp.ss$Partype=='rowphi'])]
  }
  names(temp.ss)[1] <- 'Component'
  rownames(temp.ss) <- temp.ss$Component
  Mcc <- cbind((col(M)*M) %*% rep(1,dim(M)[2]), rep(1,dim(M)[1]))
  #  x <- as.vector(t(matrix(1:231, nrow=77, ncol=3)))
  #  M2 <- M[x,]
  temp.ss <- temp.ss[order,]
  Mvcm <- M[order,]
  return(list(gammas=temp.ss,Mcc=Mcc))
}

faidesum.mfxlm <- function(obj.asr, data.df, ide.k=1, Gfac='name',Efac='location', trim=FALSE) {
  ide.sum <- list()
  snam <- as.character(levels(data.df[[Efac]]))
```

```
  gnam <- as.character(levels(data.df[[Gfac]]))
  ss <- summary(obj.asr)$varcomp
  ss$param <- row.names(ss)
  # get all variance parameters associated with Gfac (rr+diag) in model
  pp <- ss[grep(Gfac,ss$param),c('component','bound','param')]
  ide.gam.temp <- loadsum.mfxlm(param=pp, snam, k=ide.k, trim)
  ide.sum$gammas <- ide.gam.temp$out.list
  # now for the blups
  cc <- obj.asr$coef$random
  vc <- obj.asr$vcoeff$random
  # fix problems with funny characters '\xa0' so can use grep
  dimnames(cc)[[1]] <- enc2native(dimnames(cc)[[1]])
  names(vc) <- enc2native(dimnames(cc)[[1]])
  cc.ide <- cc[grep(Gfac,dimnames(cc)[[1]]),1]
  vc.ide <- vc[grep(Gfac,names(vc))]
  ide.blup.temp <- blupsum.mfxlm(cc.ide, vc.ide, ide.k, gnam, snam, data.df, Gfac, Efac,
                               ide.gam.temp, Excludevm=NULL, trim)
  ide.sum$blups <- ide.blup.temp$blup.df
  ide.sum$scores <- ide.blup.temp$score.df
  # add accuracies to CVE blups and re-name regblup as CVE
  names(ide.sum$blups)[4:5] <- c('CVE','seCVE')
  Lam <- ide.sum$gammas$`rotated loads`
  Dmat <- ide.sum$gammas$Dmat
  gvar <- diag(Lam%*%Dmat%*%t(Lam))
  names(gvar) <- as.character(dimnames(Lam)[[1]])
  blups <- ide.sum$blups
  blups$gvar <- gvar[blups[[Efac]]]
  ide.sum$blups$accCVE <- sqrt(1 - ide.sum$blups$seCVE^2/blups$gvar)
  ide.sum
}

loadsum.mfxlm <- function(param, snam, k, trim) {
  nsite <- length(snam)
  # get any GxE parameters with .var in name. this will include dummy specific variances
  # (all fixed to 0) from rr term and actual specific variances from diag term
  #  psi <- param[grep('.var',param$param),]
  # get rid of specific variances associated with rr term
  psi <- param[grep('rr\\(',param$param,invert=TRUE),'component']
  # 24/10/20 extra to allow trimming of LOF
  if (trim) {
    ss.psi <- param[grep('rr\\(',param$param,invert=TRUE),]
    # re-order so in alphabetical order of environments (assumes snam is alpha order)
    # ss.psi will have names of at(Efac, ??)... where ?? is an element of snam
    # so alpha order of names will put in alpha order of snam
    ss.psi <- ss.psi[order(ss.psi$param),]
    psi <- ss.psi$component
  }
  names(psi) <- snam
  Lam <- matrix(NA, nrow=nsite, ncol=k)
  Lam[,1] <- param[grep('.fa1',param$param),]$component
  if (k > 1) {
    for (i in 2:k) {
      Lam[,i] <- param[grep(paste('.fa',i,sep=''),param$param),]$component
    }
  }
  Lam.orig <- Lam
  svdL <- svd.mfxlm(Lam.orig)
  Lam <- svdL$u
  Dmat <- svdL$d2
```

```r
  dimnames(Lam) <- list(snam, paste("fac", 1:k, sep = "_"))
  dimnames(Dmat) <- list(paste("fac", 1:k, sep = "_"), paste("fac", 1:k, sep = "_"))
  Gmat <- Lam%*%Dmat%*%t(Lam) + diag(psi)
  Cmat <- cov2cor(Gmat)
  paf.site <- matrix(0, nrow = nsite, ncol = k)
  dimnames(paf.site) <- list(snam, paste("fac", 1:k, sep = "_"))
  for (i in 1:k) {
    paf.site[, i] <- 100 * (Dmat[i,i] * diag(Lam[, i] %*% t(Lam[, i])))/diag(Gmat)
  }
  if (k > 1) {
    all <- 100 * diag(Lam %*% Dmat %*% t(Lam))/diag(Gmat)
    paf.site <- cbind(paf.site, all)
  }
  paf.mod <- 100 * sum(diag(Lam %*% Dmat %*% t(Lam)))/sum(diag(Gmat))
  paf.fac <- 100*diag(Dmat)/sum(diag(Gmat))
  dd <- 1/sqrt(diag(Gmat))
  Lamc <- diag(dd) %*% Lam
  dimnames(Lamc) <- dimnames(Lam.orig) <- dimnames(Lam)
  out.list <- list(Gmat, Cmat, paf.site, paf.fac, paf.mod, Lam, psi, Lamc, Lam.orig, Dmat)
  names(out.list) <- c("Gmat", "Cmat", "site %vaf", "factor %vaf", "total %vaf",
                       "rotated loads", "specific var", "rotated loads - c", "raw loads", "Dmat")
  list(out.list=out.list, svdL=svdL)
}

svd.mfxlm <- function(Lam) {
  # note can use this for nfac=1. make sure Lam is a matrix first
  svdL <- svd(Lam)
  u1neg <- 100*length(svdL$u[,1][svdL$u[,1]<0])/dim(Lam)[[1]]
  if (u1neg > 50) {
    svdL$u <- -1*svdL$u
    svdL$v <- -1*svdL$v
  }
  # make svdL$d a diagonal matrix here - especially important
  # for nfac=1 case - also save squared singular values
  svdL$d2 <- diag(svdL$d^2,nrow=length(svdL$d))
  svdL$d <- diag(svdL$d,nrow=length(svdL$d))
  svdL
}

blupsum.mfxlm <- function(coef, vcoef, nfac, gnam.ped, snam, data.df, Gfac, Efac, gam.temp, Excludevm, trim) {
  nsite <- length(snam)
  ngeno.ped <- length(gnam.ped)  # genos in pedigree
  # get regblups and pev directly from rr term
  coef.all <- coef[grep('rr\\(',names(coef))]
  # check length
  if (length(coef.all)!=(nsite+nfac)*ngeno.ped) {
    cat('\nWarning: rr BLUPs are incorrect length')
  }
  coef.rr <- coef.all[1:(nsite*ngeno.ped)] # take first nsite x ngeno.ped blups. rest are scores
  vcoef.rr <- vcoef[grep('rr\\(',names(vcoef))][1:(nsite*ngeno.ped)]
  # get delta blups from diag term (ie. not rr term)
  # 24/10/20 only do this if not using trim.
  if (trim==FALSE) {
    coef.dd <- coef[grep('rr\\(',names(coef),invert=TRUE)]
    if(!is.null(Excludevm)) coef.dd <- coef.dd[grep(Excludevm,names(coef.dd),invert=TRUE)] # NEW LINE!!!
    # check length
    if (length(coef.dd)!=nsite*ngeno.ped) {
      cat('\nWarning: delta BLUPs are incorrect length')
    }
```

```
  }
  if (trim==TRUE) {coef.dd <- rep(NA, length(coef.rr))}
  blup.df <- data.frame(blup=coef.rr+coef.dd, regblup=coef.rr, seregblup=sqrt(vcoef.rr))
  blup.df[[Gfac]] <- rep(gnam.ped,nsite)
  blup.df[[Efac]] <- rep(snam,each=ngeno.ped)
  blup.df$GE <- paste(blup.df[[Gfac]],blup.df[[Efac]],sep='x')
  pp <- table(data.df[[Gfac]],data.df[[Efac]])
  gnam <- dimnames(pp)[[1]]
  ngeno <- length(gnam)
  reps <- as.numeric(pp)
  names(reps) <- paste(rep(gnam,nsite),rep(snam,each=ngeno),sep='x')
  blup.df$reps <- reps[blup.df$GE]
  blup.df$reps[is.na(blup.df$reps)] <- 0
  blup.df <- blup.df[,c(5,4,1,2,3,7)]
  # now for scores ...
  scores <- coef.all[(nsite*ngeno.ped+1):length(coef.all)]
  score.mat <- matrix(scores, ncol = nfac)
  score.mat <- score.mat %*% gam.temp$svdL$v %*% gam.temp$svdL$d
  score.df <- data.frame(score=as.vector(score.mat))
  score.df$comp <- paste('Comp',rep(1:nfac,each=ngeno.ped),sep='')
  score.df[[Gfac]] <- rep(gnam.ped,nfac)
  score.df <- score.df[,c(2,3,1)]
  # check on regblups.
  regblup <- as.vector(score.mat %*% t(gam.temp$out.list$'rotated loads'))
  pcdiff <- 100*abs((blup.df$regblup-regblup)/regblup)
  pcdiff[is.na(pcdiff)] <- 0
  mm <- mean(pcdiff)
  #  if (mm > 0.5) {
  cat('\nMean % difference between regblup coeffs and (rotated) loads * scores', mm)
  #  }
  list(blup.df=blup.df, score.df=score.df)
}

update.mfxlm <- function(obj,sv,diff,boundfix=TRUE) {
  ##########
  ## Function to update from one model to another
  ## should work for any general problem with vm and ide
  ## companion to pikk.update.coloc
  ## start with R gammas
  ##
  ## we have a pikk.coloc
  ## eg move from fak no spatial to fak spatial
  ## input obj - target asreml object for spatial terms can be fa or diag,
  ## input sv - template for new model
  ## input - optional for colocated trials as the output from vcc
  ##
  vparams.tab <- sv$vparameters.table
  oldgams <- summary(obj)$varcomp
  oldgams$Component <- dimnames(oldgams)[[1]]
  oldvpars <- summary(obj,vparameters=TRUE)$vparameters
  rpar.rows <- grep('\\!R',
                  substring(vparams.tab$Component,nchar(vparams.tab$Component)-1,
                            nchar(vparams.tab$Component)))
  rpar.rows <- seq(from=min(rpar.rows),to=nrow(vparams.tab))
  vv.vcc <- vparams.tab[rpar.rows,]
  temp <- merge(vv.vcc,oldgams,by='Component')
  temp <- temp[,c('Component','component','Constraint')]
  names(temp)[2] <- 'Value'
  R.sv <- temp
```

```
  #################
  # now do the genetic and non-genetic G
  # drop R terms which are in co-located
  # I will need another hook if not colocated
  # best in another function
  # one less argument
  # the way forward is to have a list which does a match on Component &
  # names(oldpars) eg here we have 'rr.*vm'
  # as the string match and then we want to replace
  # fa1 and fa2 in component with what we provide
  # e.g.
  # diff=list('rr.*vm'=list('fa1'=NULL,'fa2'=c(0,rep(.02,nsite-1))))
  # as the argument to the function
  # this takes the diff and uses the elements to replace NAs in the component in the merge
  # which are those variance parameters which are in terms which have changed
  # replace them with oldgams if is.null, else replace them with values in the
  # named vector where the name of the vector is used to identify the variance parameters in the
  # term to be replaced!!
  temp <- subset(vparams.tab,!is.element(Component,vv.vcc$Component))
  temp$index <- 1:nrow(temp)
  temp <- merge(temp,oldgams,by='Component',all.x = TRUE)
  temp <- temp[order(temp$index),]
  G.sv <- subset(temp,!is.na(component)) # this stores those with a match
  ####################
  # now process those which are not a match and have NA in component
  #
  temp <- subset(temp,is.na(component))
  cat('Number of non matched variance parameters',nrow(temp))
  #  diff <-list('rr.*vm'=list('fa1'=NULL,'fa2'=c(0,rep(.02,nsite-1))))
  for(tt in names(diff)){
    cat('...and those which match the diff name', tt,' is',
        nrow(temp[grep(pattern = tt,x=temp$Component),]),'\n')
    if(any(sapply(diff[[tt]],is.null))) thisgam <- oldvpars[[grep(pattern = tt,x=names(oldvpars))]]
    for(pp in names(diff[[tt]])){
      xx <- diff[[tt]][[pp]]
      print(xx)
      cat(paste(tt,pp,sep='.*'))
      if(is.null(xx)) {
        temp[grep(pattern=paste(tt,pp,sep='.*'),x=temp$Component),'component'] <-
          thisgam[grep(pattern=paste(tt,pp,sep='.*'),x=names(thisgam))]
      } else {
        temp[grep(pattern=paste(tt,pp,sep='.*'),x=temp$Component),'component'] <- xx
      }
    }
  }
  temp[is.na(temp$component),'component'] <- temp[is.na(temp$component),'Value']
  G.sv <- rbind(G.sv,temp)
  G.sv <- G.sv[,c('Component','component','Constraint')]
  names(G.sv)[2] <- 'Value'
  ################
  ## now tidy up the starting values (Values) which are too small for Constraint=='P'
  ## in G level terms
  if(boundfix) G.sv$Value[G.sv$Constraint=='P' & G.sv$Value<1e-5] <- 1e-4
  return(list(G.sv=G.sv,R.sv=R.sv))
}
```

2 Factor Analytic Linear Mixed Model and summary in ASReml-R

```
# Load the package ASReml-R
# Note that this software can be obtained from https://www.vsni.co.uk/software/asreml-r
require(asreml)


# We run FALMM in ASReml-R by commencing with an FA1 model then using the resultant variance
# parameter estimates as starting values for the FA2 model and so on.
# In the followng code we have already fitted FA1, FA2 and FA3 models and are using the FA3
# model to obtain starting values for the final model fitted, that is an FA4.
# We obtain starting values by first using our in-house summary function
# (called faidesum.mfxlm) on the asreml object for the run of the FA3
# model (object is called fa3.asr):

temp.sum <- faidesum.mfxlm (fa3.asr, a4.df, ide.k = 3, Gfac = "name", Efac = "Env", trim = FALSE)
# here the data-frame is called a4.df; we have fitted ide.k=3 factors in the model;
# the name of the variety factor in the data-frame is "name"; the name of the
# environment factor in the data-frame is "Env"


# FA4 ---------------------------------------------------------------------
# first set up object containing (default) starting values

fa4.sv <- asreml(yield~Env,
                 random=~rr(Env, 4):name + diag(Env):name +
                   at(Env,me.nam):expt + at(Env, cb.namf):expt:Cblock +
                   at(Env,rb.namf):expt:Rblock + at(Env,col.namf):expt:Column +
                   at(Env,row.namf):expt:Row,
                 residual=~dsum(~ar1(Column):ar1(Row) + id(Column):ar1(Row) + id(Column):id(Row) +
                                  ar1(Column):id(Row)|expt,
                                levels=list(ar1ar1.namf,idar1.namf,idid.namf,ar1id.namf)),
                 data=a4.df,na.action = na.method(x='include'), start.values = TRUE)
# This set-up is for a model that fits an FA4 where the FA variance structure is
# split into two parts corresponding to the common effects (rr(Env, 4):name)
# and specific effects (diag(Env):name). There are a number of peripheral effects
# and there are spatial models for the residuals (see table 3 in paper).

# now use in-house functions to compute starting values etc.

# First we need a constraint matrix that will constrain the spatial correlation
# and residual parametes for all the experiments in an environment to be the same.
# Note that this bit of code is only necessary when there are co-located trials
# in an environment and when their row and column numbers are indexed within trials
# rather than the environment as a whole.

vv <- vccmake.mfxlm(obj=fa4.sv,data=a4.df,Rownam = "Row!cor", Colnam='Column!cor',Resnam='!R$',
                    Envnam = 'Env', Trialnam='expt')

# Now we work out starting values that we take from the fit of the FA3 model:
mydiff <- list('rr'=list('fa1'=temp.sum$gammas$`raw loads`[,1],'fa2'=temp.sum$gammas$`raw loads`[,2],
                         'fa3'=temp.sum$gammas$`raw loads`[,3],'fa4'=c(rep(0,3),rep(0.01,(ne-3)))))
gam <- update.mfxlm(obj=fa3.asr, sv=fa4.sv, diff=mydiff, boundfix=TRUE)

# Finally we run the FA4 model using these starting values and the constraint matrix
fa4.asr <- asreml(yield~Env,
                  random=~rr(Env, 4):name + diag(Env):name +
                    at(Env,me.nam):expt + at(Env, cb.namf):expt:Cblock +
                    at(Env,rb.namf):expt:Rblock + at(Env,col.namf):expt:Column +
                    at(Env,row.namf):expt:Row,
```

```r
                  residual=~dsum(~ar1(Column):ar1(Row) + id(Column):ar1(Row) + id(Column):id(Row) +
                                 ar1(Column):id(Row)|expt,
                              levels=list(ar1ar1.namf,idar1.namf,idid.namf,ar1id.namf)),
                  vcc=vv$Mcc, R.param=gam$R.sv, G.param=gam$G.sv,
                  data=a4.df, na.action = na.method(x='include'))

# once the model has converged (it may require some updates) we run
# our in-house summary function on the asreml object fa4.asr:
fa4.sum <- faidesum.mfxlm (fa4.asr, a4.df, ide.k = 4, Gfac = "name", Efac = "Env", trim = FALSE)
# this is a list with 3 elements
names(fa4.sum)
# "gammas" "blups"  "scores"
# The first of these is also a list with elements:
names(fa4.sum$gammas)
#[1] "Gmat"               "Cmat"               "site %vaf"          "factor %vaf"        "total %vaf"
#[6] "rotated loads"      "specific var"       "rotated loads - c" "raw loads"          "Dmat"
# The others (fa4.sum%blups and fa4.sum$scores) are data-frames holding the
# EBLUPs of the CVEs and variety scores respectively.

# now some code to work out the iClasses for the environments:
# (so as per table 4 in the manuscript)
lam.df <- as.data.frame(fa4.sum$gammas$`rotated loads`)
names(lam.df) <- c('lam1','lam2','lam3','lam4')
lam.df$vaf <- fa4.sum$gammas$`site %vaf`[,'all']
lam.df$Env <- row.names(lam.df)

# create +/- split of each factor
# lam.df$Sfac1 <- factor(lam.df$lam1>0,labels = c('n','p'))
# no negative in first factor so can't form Sfac1 like this
lam.df$Sfac1 <- 'p'
lam.df$Sfac2 <- factor(lam.df$lam2>0,labels = c('n','p'))
lam.df$Sfac3 <- factor(lam.df$lam3>0,labels = c('n','p'))
lam.df$Sfac4 <- factor(lam.df$lam4>0,labels = c('n','p'))
# create iCclass factor by pasting these levels
lam.df$iClass <- factor(paste(lam.df$Sfac1,lam.df$Sfac2,lam.df$Sfac3,lam.df$Sfac4,sep=''))
table(lam.df$iClass)
#pnnn pnnp pnpn pnpp ppnn ppnp pppn pppp
#   8    6    8    5   10    8   17   11

# correlation matrix for heatmap of figure 1
Cmat.beta <- fa4.sum$gammas$`rotated loads`%*%fa4.sum$gammas$Dmat%*%t(fa4.sum$gammas$`rotated loads`)
Cmat.beta <- cov2cor(Cmat.beta)
diag(Cmat.beta) <- NA

# variety scores
sc.df <- as.data.frame(with(fa4.sum$scores,tapply(score,list(name,comp),mean)))
names(sc.df) <- c('fac1','fac2','fac3','fac4')
sc.df$name <- row.names(sc.df)


# iClassOP for all varieties
nc <- length(unique(lam.df$iClass))
nk <- 4 # number of factors in model
temp.df <- merge(fa4.sum$blups,lam.df,by='Env')
temp.df <- merge(temp.df,sc.df,by='name')
# mean loadings values at which OP predictions are made (as per table 5)
(lambar <- apply(lam.df[,1:nk],2,function(x,f) tapply(x,f,mean), f=lam.df$iClass))
temp.df$name <- factor(temp.df$name)
temp.lst <- split(x = temp.df, f = temp.df$iClass)
```

```r
imeans.lst <- list()
# iClass OP calculated as mean of CVEs
for(i in names(temp.lst)) {
  imeans.lst[[i]] <- with(temp.lst[[i]],tapply(CVE,name,mean))
}
iClassOP <- matrix(unlist(imeans.lst),ncol=nc)
dimnames(iClassOP) <- list(levels(temp.df$name),names(temp.lst))
```
